# Case Report: Adrenal Pathology Findings in Severe COVID-19: An Autopsy Study

**DOI:** 10.4269/ajtmh.20-0787

**Published:** 2020-08-31

**Authors:** Monique Freire Santana, Mayla Gabriela Silva Borba, Djane Clarys Baía-da-Silva, Fernando Val, Márcia Almeida Araújo Alexandre, Jose Diego Brito-Sousa, Gisely Cardoso Melo, Marcos Vinícius Oliveira Queiroga, Maria Eduarda Leão Farias, Cecília Cunha Camilo, Felipe Gomes Naveca, Mariana Simão Xavier, Wuelton Marcelo Monteiro, Guilherme Augusto Pivoto João, Ludhmila Abrahão Hajjar, Jaume Ordi, Marcus Vinícius Guimarães Lacerda, Luiz Carlos Lima Ferreira

**Affiliations:** 1 Departamento de Ensino e Pesquisa, Fundação de Medicina Tropical Dr. Heitor Vieira Dourado, Manaus, Brazil;; 2 Programa de Pós-Graduação em Medicina Tropical, Universidade do Estado do Amazonas, Manaus, Brazil;; 3 Departamento de Ensino e Pesquisa, Fundação Centro de Controle de Oncologia do Estado do Amazonas – FCECON, Manaus, Brazil;; 4 Hospital e Pronto Socorro Delphina Rinaldi Abdel Aziz, Manaus, Brazil;; 5 Fundação Oswaldo Cruz, Instituto de Pesquisas Leônidas and Maria Deane, Manaus, Brazil;; 6 CDL – Laboratórios Santos e Vidal, Manaus, Brazil;; 7 Universidade de São Paulo – USP, São Paulo, Brazil;; 8 Hospital Clínic de Barcelona, Barcelona, Spain

## Abstract

Although high mortality has been reported in many COVID-19 studies, very limited postmortem information from complete autopsies is available. We report the findings in the adrenal glands in 28 autopsies with confirmed SARS-CoV-2 infection. Microscopic lesions were identified in the adrenal glands in 12/28 patients (46%). Seven cases showed necrosis, generally ischemic; four showed cortical lipid degeneration; two showed hemorrhage; and one unspecific focal adrenalitis. Vascular thrombosis in one patient and focal inflammation in association with other findings in three patients were observed. No case presented adrenal insufficiency. In conclusion, adrenal lesions are frequent in patients with severe COVID-19. The lesions are mild but could contribute to the lethal outcome.

The current COVID-19 pandemic caused by SARS-CoV-2 has lasted for about 6 months in more than 10 million cases worldwide after the first cases were detected in China in late 2019. In mild cases, the infection is usually self-limited and does not require specialized care. Patients with severe COVID-19, however, often require intensive care because of respiratory failure.^1^ Exacerbated inflammation due to a cytokine storm leads to rapid multiple organ failure and fatal damage to the lungs, kidneys, and heart,^2^ and has been shown as a key pathogenetic mechanism in these severe COVID-19 cases.

This intense inflammatory process is commonly associated with the activation of coagulation.^3^ This phenomenon is also observed in infections by *Neisseria meningitidis*, *Mycobacterium tuberculosis*, *Cytomegalovirus*, Ebola, and *Histoplasma capsulatum*, and frequently evolves to adrenal injury due to hemorrhage and/or thrombosis.^4^ However, adrenal complications have not been reported in COVID-19 patients so far.

Complete autopsies were performed on 28 deceased patients with SARS-CoV-2 infection confirmed by reverse transcription quantitative polymerase chain reaction^5^ of nasopharyngeal/oropharyngeal swab samples from the referral hospital for severe COVID-19 infections in Manaus (Hospital e Pronto Socorro Delphina Rinaldi Abdel Aziz) in the Western Brazilian Amazon. Signed informed consent was obtained from the legal representatives in all cases. All patients were examined between March and May 2020. Autopsies were conducted in the same hospital by a single pathologist, with the help of trained technicians, under strict biosafety rules. Specimens were fixed in neutral buffered formalin, paraffin embedded, and routinely stained with hematoxylin and eosin. All cases were examined by two different pathologists, blind to each other. In case of discrepancy, an adjudication meeting was held, and a consensus was reached. Clinical, macroscopic, and microscopic data were systematically registered in Research Electronic Data Capture standard forms.

The autopsy study showed adrenal lesions in 12 of the 28 patients (42.9%) who died with severe COVID-19 (Table 1; see Figure 1 for most representative macroscopic and microscopic images of adrenal involvement in COVID-19 in five patients). Seven cases showed necrosis, which was generally ischemic necrosis (four cases). Four cases showed cortical lipid degeneration, two showed hemorrhage, and one unspecific focal adrenalitis. Focal inflammation was observed in association with other findings in three patients, whereas vascular thrombosis was seen in one patient. Overall, focal inflammation was identified in 4/28 patients (14%). In one of the patients, adrenal cortical carcinoma was detected as an incidental finding. Most of these patients were male (10/12 or 83.3%), aged 34–88 years, and 10 (83.3%) presented known comorbidities including alcoholism, smoking, and obesity (Table 1).

**Table 1:** Demographics and plasma cortisol of patients with adrenal gland microscopic findings

Patient	Age/gender	Comorbidities	Days of disease before death	Cortisol 24–48 hours before death (μg/dL)	Adrenal glands
1	57/M	–	10	NA	Ischemic necrosis
2	34/M	Alcoholism and smoking	17	54.2	Hemorrhagic necrosis
3	66/M	Hypertension and smoking	11	43.5	Focal ischemic necrosis with thrombus
4	65/M	Alcoholism and Parkinson	55	65.8	Cortical lipid degeneration and adrenocortical carcinoma
5	88/M	Coronary insufficiency	12	23.5	Ischemic necrosis
6	65/F	–	10	67.2	Cortical lipid degeneration and focal adrenalitis
7	48/M	Obesity	22	14.6	Ischemic necrosis
8	55/M	Smoking	19	73.2	Hemorrhagic necrosis
9	50/F	Obesity and chronic kidney diseases	12	41.5	Cortical lipid degeneration
10	35/M	Obesity and alcoholism	21	41.9	Cortical lipid degeneration and focal adrenalitis
11	52/M	Diabetes mellitus, alcoholism, smoking, and gout	12	80.7	Focal necrosis and focal adrenalitis
12	70/M	Alcoholism and smoking	23	70.1	Focal adrenalitis

F = female; M = male; NA = not available. Obesity: body mass index > 30; cortisol reference levels: 4.3–22.4 μg/dL. Focal adrenalitis: unspecific presence of lymphocytes; Ischemic necrosis: more than ∼40% of the gland was compromised.

**Figure 1.**
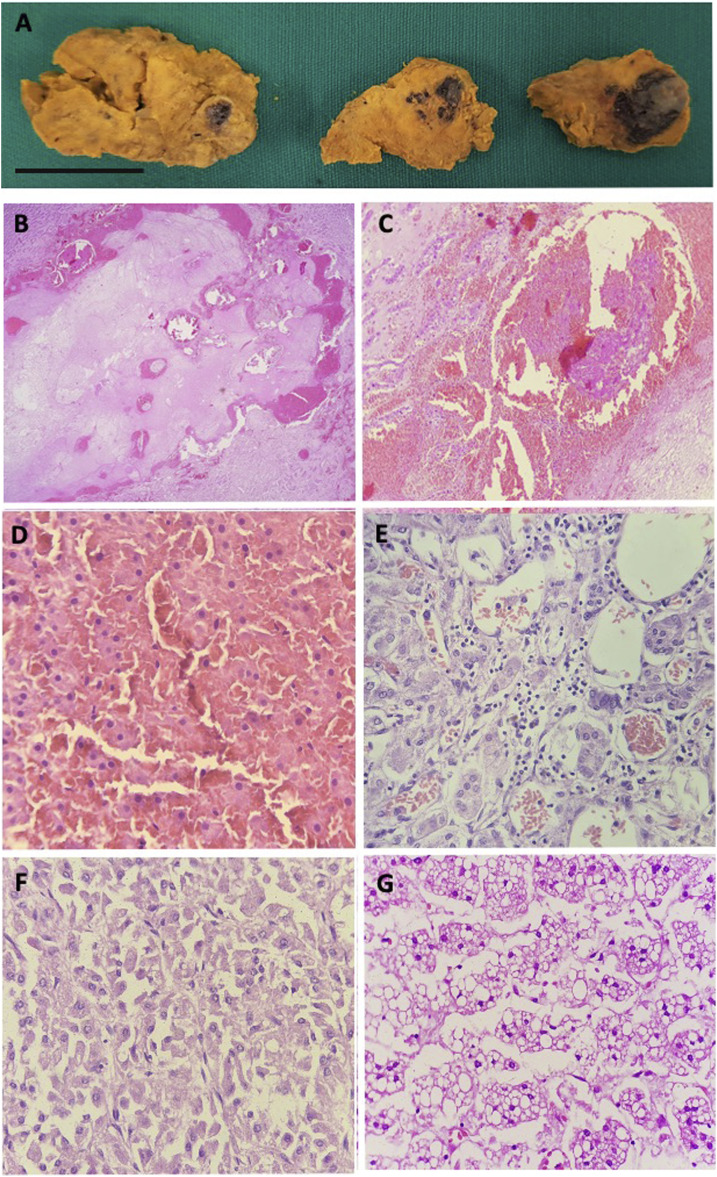
Adrenal pathology autopsy findings in five severe COVID-19 patients. Enlarged right adrenal gland with extensive areas of hemorrhagic necrosis (**A**) (patient 8), bar = 2 cm. Histologic section of the adrenal with extensive adrenal hemorrhage (**B**) (patient 8), thrombi fibrin (**C**) (patient 8), hemorrhagic necrosis (**D**) (patient 2), focal adrenalitis (**E**) (patient 12), and adrenocortical carcinoma (**F**) (patient 4). Adrenal cortex with diffuse and severe cortical vacuolization of macro- and microvacuolar lipidic deposits (**G**) (patient 9). Hematoxylin–eosin–stained tissues observed under ×400 original magnification.

After the autopsy findings, stored samples of plasma collected 1 or 2 days before death were sent for cortisol measurement, by quimioluminescence. No cortisol was less than 10 μg/dL (Table 1), which is a reliable and widely available laboratorial marker of adrenal insufficiency in critical patients in practice.^6^ All patients had been treated with wide-spectrum antibiotics and vasopressors (mostly norepinephrine) at some stage of the hospitalization, and only patients 11 and 12 used corticoid therapy (hydrocortisone).

Because of biosafety concerns, very few pathology laboratories worldwide have been able to perform full autopsies of COVID-19 patients. Therefore, most of the series are based on minimally invasive autopsies (MIAs),^7–9^ in which adrenal glands are not generally accessible. However, MIAs can be useful for COVID-19 death surveillance purposes and the study of more accessible target organs, such as lungs. Only one series has examined the adrenal glands in five cases; this study reported only minor vascular damage in the adrenal glands.^10^ In this series, the fibrinoid hyaline vasculopathy in peri-adrenal vessels found was directly related to atherosclerosis in patients with systemic arterial hypertension and diabetes mellitus, and, consequently, considered as more related to the preexisting disease than to the viral infection. By contrast, in the present series, the adrenal lesions were likely caused by SARS-CoV-2 infection.

In our study, adrenal lesions were frequently observed in patients who died with severe COVID-19 (46%). The detected lesions (necrosis, cortical lipid degeneration, hemorrhage, and focal inflammation) were possibly directly linked to the viral infection, as no associated lesions were identified. However, microthrombi were identified in one patient as opposed to pulmonary microthrombi reported as a common finding in COVID-19.^11^ One major limitation of our series was the lack of viral identification in the adrenal glands, making it possible that the findings could also be explained by another nonspecific systemic end-stage fatal disease. In all cases, however, the major cause of death on postmortem examination was attributed to SARS-CoV-2 infection, and extensive lung commitment was the major complication directly related to death.

In four of our COVID-19 cases, the adrenal cells presented cortical lipid degeneration, characterized by the accumulation of clear vacuoles within cortical cells. Cortical lipid degeneration (vacuolization) has been shown to occur as a spontaneous, age-related change or as secondary to stress from various causes, such as drugs and toxins, or infection.^12^ In stressful situations, adrenal receptors are activated and induce the synthesis of steroidogenic enzymes as well as cholesterol production and transportation (which can potentially accumulate), culminating in the production and release of glucocorticoids.^13^

Corticosteroids have a potential role in controlling the host inflammatory response, potently inhibiting cytokine production and restoring inappropriately low endogenous levels of cortisol.^14^ Adrenal damage compromises the regulation of inflammation and may be harmful. Some infectious agents affect the adrenal gland, leading to refractory shock, the classical Waterhouse–Friderichsen syndrome.^15^

In critically ill patients, catecholamines released in the procoagulant environment may cause vasoconstriction in adrenal veins, resulting in venous thrombosis and adrenal necrosis.^16^ The use of exogenous vasopressors may enhance this process. Of 78 patients with adrenal necrosis, 41% had thrombosis of the main adrenal vein and the capsular veins.^17^ Venous thrombosis can occur in most of the cases in patients with severe infection, especially in the respiratory tract,^18^ and bacterial infections.^19^ Unilateral adrenal hemorrhage and necrosis, features identified in most of the reported patients, may result from direct damage to the adrenal or blood supply.

The moderate adrenal injuries observed in this series suggest a need for further full autopsy studies to clarify their role in the mechanism leading to death in these patients. Therefore, corticosteroid efficacy against COVID-19, as recently shown (Recovery trial),^20^ is probably fundamented on its pulmonary anti-inflammatory properties.
